# The course of bipolar disorder in rural India

**DOI:** 10.4103/0019-5545.31559

**Published:** 2006

**Authors:** Mohit P. Chopra, K.V. Kishore Kumar, D.K. Subbakrishna, Sanjeev Jain, R. Srinivasa Murthy

**Affiliations:** * Assistant Professor, Department of Psychiatry, National Institute of Mental Health and Neuro Sciences (NIMHANS), Hosur Road, Bangalore 560029, Karnataka; ** Senior Resident, Department of Psychiatry, National Institute of Mental Health and Neuro Sciences (NIMHANS), Hosur Road, Bangalore 560029, Karnataka; **** Professor, Department of Psychiatry, National Institute of Mental Health and Neuro Sciences (NIMHANS), Hosur Road, Bangalore 560029, Karnataka; ***** Professor, Department of Psychiatry, National Institute of Mental Health and Neuro Sciences (NIMHANS), Hosur Road, Bangalore 560029, Karnataka; *** Professor, Department of Bio-Statistics, National Institute of Mental Health and Neuro Sciences (NIMHANS), Hosur Road, Bangalore 560029, Karnataka

**Keywords:** Bipolar disorder, mania, longitudinal follow-up, rural

## Abstract

**Aim:**

To examine the naturalistic course of bipolar disorder in a rural, community-based, partially treated cohort.

**Methods:**

All patients diagnosed with bipolar disorder during an epidemiological survey (*n*=34) in a rural area in India were followed longitudinally using standardized instruments, and the life-chart method used to examine their course.

**Results:**

Seven (26%) of the 27 patients evaluated directly had not received any treatment whatsoever. Four patients (15%) had experienced rapid-cycling at some time; patients without rapid-cycling had experienced a mean 0.22 episodes/year. Episodes of mania accounted for 72% of all episodes. None of the variables examined appeared to predict the total number of episodes experienced by individual patients, although rapid-cycling occurred significantly more often if the patients had not received any psychopharmacological treatment.

**Conclusions:**

A mania-predominant course was observed in this small cohort, similar to reports from other developing countries.

## INTROUCTION

Several longitudinal studies show that bipolar disorder is characterized by frequent recurrences^1^–^3^ and multiple depressive episodes.^4^ A limitation is that these studies were conducted at specialist centres. Only few longitudinal evalua-tions of bipolar patients have been conducted in developing countries^5^–^7^ and they indicate a higher proportion of manic episodes. Longitudinal studies carried out in India have supported these findings^8^,^9^ but also followed patients at specialized centres.

The specific aim of this report is to evaluate the course of bipolar disorder in a rural community setting. Data were obtained about the course of bipolar disorder in a cohort of patients who had previously been diagnosed during an epidemiological survey in southern India,^10^ and examined potential determinants of outcome. These data provided a unique opportunity to evaluate the course among subjects, the majority of whom had not accepted prophylactic treatment, even though it had been offered to them.

## METHOD

### Background

India's National Institute of Mental Health and Neuro Sciences (NIMHANS) has adopted the Sakalwara Primary Health Center (PHC) in a rural area near the city of Bangalore. An epidemiological survey of major psychiatric disorders was conducted in this area.^10^ An earlier publication describes three methods of case-detection that were used to screen for cases in this population.^11^ Cases diagnosed with a psychiatric con-dition, using the ICD-10^12^ system, during the survey were placed under follow-up care of the Sakalwara PHC staff, which consisted of psychiatrists, psychiatric nurses and social workers. Typically, patients received home visits by staff members at about 6-month intervals, and were offered psychotropic medications as their individual clinical condition warranted. They were free to accept or refuse the treatment, except when their behavior put them at risk of harm, when they could be involuntarily committed for management.

### Subjects and procedure

Thirty-four patients met the criteria for bipolar disorder (formerly manic–depressive psychosis, manic or circular) during the epidemiological survey.^10^ During 1997, about 8 years after the epidemiological survey, a follow-up evaluation of these patients was conducted over a 6-month period at their place of residence. This involved frequent travel to remote villages through difficult terrain. The attempt was made to trace any patients who had migrated from their original residence. The research review committee of the NIMHANS had approved the protocol and study consent forms, and no difficulty was experienced with consent for assessment, mainly because of the long-standing relationship between the patients and the PHC staff.

Patients were evaluated using the *Schedule for Affective Disorders and Schizophrenia—Lifetime version (SADS-L)*^13^ and the *Longitudinal Interval Follow-up Evaluations (LIFE).*^14^ Information from multiple sources was integrated during the follow-up evaluation, including information from direct interview of patients and other collateral sources. The evaluation was considered complete when we were able to successfully conduct a direct face-to-face interview with the patient and at least one other family member who had observed the patient for a significant time during the follow-up period. Since patients in India often report somatic symptoms rather than complaints of depressed mood, we were particularly careful during the SADS-L interview to screen for missed depressive symptoms or episodes.

Multiple visits were made to ensure complete data collection. The final data were used to construct a longitudinal course of the patient's illness using the Life Chart Method.^15^ The research team then reviewed the life-chart with the patient and family, and any discrepancies were resolved using a consensus approach. The entire process took about 2–3 days per patient to complete. It was not possible to conduct frequent structured follow-up evaluations for financial and logistic reasons, and hence this limitation was overcome by being as thorough and complete as possible in the data gathering effort.

### Statistical analysis

Data was analysed using the SPSS v11.5 program. Descriptive analyses included means and standard deviations for continuous variables and frequencies for categorical variables. The results are presented in a tabular form. The data was also analysed to identify potential correlates of the life-course, and included a logistic regression analysis using the occurrence of rapid-cycling as the dichotomous dependent variable and the administration of any psychopharmacologic treatment as the predictor variable. Significant findings are presented due to space limitations.

## RESULTS

Of the 34 bipolar patients, 2 patients could not be traced as they had migrated from the catchment area. Four patients had died from natural causes.There were no deaths from suicide. Hence, follow-up information was obtained for 32 of the 34 patients (94%) in the original cohort, and direct interviews were carried out with 28 patients. One patient was re-diagnosed as having an Organic Mood Disorder (ICD-10).^12^

Ten men and 17 women, with a mean (±SD) age of 48 (±14.2, range: 25–77) years, were currently alive. Twenty-five (93%) patients had <7 years of education, 24 (89%) were from the lower socioeconomic strata and 26 (96%) had been married at least at some time. The mean duration of follow-up was 8.45 (±0.27, range: 8.04–8.88) years. Table 1 describes details about the onset, course, and the duration of episodes and cycles. Treatment received by these patients had been largely intermittent (Table 2).

**Table 1:** Details of the course of bipolar disorder in the cohort (*n*=27)

Variable	Mean + SD	Range
Age at onset (years)	27.7 ± 9.1	14–49
Duration of illness (years)	20.2 ± 10.7	9–52
Episode details		
Number of episodes*	4.4 ± 3.4	1–12
Duration of (weeks)		
Manic episode	13 ± 15.4	2–91
Depressive episode	18 ± 23.4	2–78
Mixed/cycling episode	10 ± 9	3–30
Cycle details		
Duration (months)		
First cycle	54 ± 80	9.5–338
Second cycle	45 ± 46	3–169
Third cycle	34 ± 27	13–78

* Excludes patients with rapid-cycling

**Table 2:** Characteristics of onset and course of bipolar disorder in the cohort (*n*=27)

Illness characteristic	*n* (%)
Number of life-time episodes	
Single episode	5 (18.5)
2–5 episodes	10 (37)
6–9 episodes	3 (11)
>10 episodes	5 (18.5)
Rapid cycling	4 (15)
Treatment details	
Currently in active treatment	6 (22)
History of previous treatment	20 (74)
—with antipsychotics only	17 (63)
—with lithium (and/or antipsychotics)	3 (11)
Never treated	7 (26)
Never hospitalized	13 (48)

### Illness onset and course

Seven patients (26%) had an onset with an episode of depression or a mixed/cycling episode. Patients without rapid cycling had a mean of 4.4 and a median of 3 major mood episodes (Table 1), resulting in a mean of 0.22 episodes/year.

However, 5 patients had experienced only a single episode of mania during the follow-up period. Eighteen patients (67%) had an illness-course characterized by many more manic than depressive episodes. Despite carefully screening for possible depressive episodes, 72% of all diagnosed episodes were those of mania.

### Episode and cycle characteristics

The mean duration for each type of episode is presented in Table 1. The mean duration of a cycle following a manic episode was 31 (±28, range: 5–169) months, the depressive cycle-length was 33 (±26.7, range: 3–72) months and the mixed episode cycle-length was 22 (±16, range: 4.5–52) months. These episode and cycle-lengths were not statistically significantly different. None of the putative baseline or treatment variables examined was found to predict the total number of episodes experienced by individual patients. However, rapid-cycling was seen to occur significantly more often among patients who had not received any psychopharmacologic intervention (OR=14.25; 95% CI: 1.16, 174.8; Wald χ^2^ = 4.3; p=0.038).

### Comorbidity

Seven patients (26%) also meet criteria for alcohol dependence (ICD-10).^12^ Six patients (22%) reported periods wherein they experienced symptoms below the threshold criteria for an episode.

## DISCUSSION

The current report contributes to the limited literature on follow-up evaluations of community-based patients in developing countries. Further, this sample offered the opportunity to study the course of bipolar disorder little affected by prophylactic pharmacotherapy. The mean number of episodes/year,^1^,^3^,^16^ and the occurrence of sub-threshold symptoms^17^ are similar to reports from developed countries.

Studies from the USA^2^,^16^ have reported that 19–28% of patients remain relapse-free over a 4–5-year period, and 11% of subjects had not relapsed during another 10-year follow-up evaluation.^3^ Five patients in this study had experienced only a single manic episode. It is possible that some bipolar patients relapse only after extended periods, and it would be worthwhile attempting to identify the determinants of such long cycles.

Consistent with other similar reports, this study also found an over-representation of mania. Interestingly, this pattern of course among bipolar patients has been reported mostly from the tropical regions, such as Nigeria,^7^ Hong-Kong^6^ and India.^8^,^9^ Is it possible that bright sunlight and a less variable day–night cycle could result in more manic episodes in the tropics? This, however, contrasts with reports from the temperate regions, where bipolar patients may experience depression for about a third of their course.^4^,^17^

Limitations of this study include the small number of patients followed, and consequently the results should be considered preliminary. Next, given logistic difficulties, we were unable to perform frequent structured evaluations. We attempted to overcome this by integrating information from multiple sources so that the data were as complete and thorough as possible. Nonetheless, in spite of all efforts, it is possible that some episodes were missed. Another possibility is that inadvertently, we examined subjects that mainly began their illness with mania, and recent evidence suggests that mood state at study entry might predict the polarity of future relapse.^18^ Finally, the results may be applicable to rural but not urban areas, where factors known to influence relapse, such as stress, are different.

The study strengths include the community-based nature of subjects. A range of relapse frequencies characterizes the course of bipolar disorder in rural south India and the influence of treatment on outcomes in a similar but larger group warrants further exploration.
